# Community Structure of Phyllosphere Bacteria in Different Cultivars of Fingered Citron (*Citrus medica* ‘Fingered’) and Their Correlations With Fragrance

**DOI:** 10.3389/fpls.2022.936252

**Published:** 2022-07-15

**Authors:** Yi Wang, Jiaqi Wu, Ping Sun, Chenfei Chen, Jiansheng Shen

**Affiliations:** Jinhua Academy of Agricultural Sciences, Jinhua, China

**Keywords:** phyllosphere bacteria, fingered citron, varieties, high-throughput sequencing, volatile organic compounds (VOCs)

## Abstract

In recent years, plant metabolomics and microbiome studies have suggested that the synthesis and secretion of plant secondary metabolites are affected by microbial-host symbiotic interactions. In this study, six varieties of fingered citron (*Citrus medica* ‘Fingered’) are sampled to study their phyllosphere bacterial communities and volatile organic compounds (VOCs). High-throughput sequencing is used to sequence the V5–V7 region of the 16S rRNA of the fingered citron phyllosphere bacteria, and the results showed that Proteobacteria, Actinobacteria, Firmicutes, and Bacteroidetes were the dominant bacterial phylum in the phyllosphere of fingered citron. There were significant differences in the phyllosphere bacteria community between XiuZhen and the remaining five varieties. The relative abundance of *Actinomycetospora* was highest in XiuZhen, and *Halomonas*, *Methylobacterium*, *Nocardioides*, and *Pseudokineococcus* were also dominant. Among the remaining varieties, *Halomonas* was the genus with the highest relative abundance, while the relative abundances of all the other genera were low. Headspace solid-phase microextraction (HS-SPME) and gas chromatography-mass spectrometry (GC-MS) were used to analyze and identify the aroma compounds of six different fingered citron, and a total of 76 aroma compounds were detected in six varieties. Pinene, geraniol, and linalool were found to be the primary VOCs that affect the aroma of fingered citron based on relative odor activity value. The correlation analysis showed 55 positive and 60 negative correlations between the phyllosphere bacterial flora and aroma compounds of fingered citron. The top 10 genera in the relative abundance were all significantly associated with aroma compounds. This study provides deep insight into the relation between bacteria and VOCs of fingered citron, and this may better explain the complexity of the analysis of bacterial and metabolic interactions.

## Introduction

Plant volatile organic compounds (VOCs) are an important component of plant secondary metabolites (PSMs) and often influence plant aroma (Johnson et al., 2016; Tian et al., 2018). VOCs are one of the most important indicators used to determine fruit quality. Fingered citron (*Citrus medica* ‘Fingered’), which belongs to Rutaceae, is a small evergreen tree. Its fruit is famous for its strong aroma and is widely used in the chemical, food, and medical industries as a raw material for extracting natural flavors. The differences in the fruit aroma of many plants, including fingered citron, are often thought to be related to variety, place of origin, and maturity (Nieuwenhuizen et al., 2015; Ji et al., 2019). Recent studies have shown that the fruit aroma is also related to the phyllosphere microbial community, such as *Methylobacterium*, *Bacillus*, and *Lactobacillus* (Nasopoulou et al., 2014; Gargallo-Garriga et al., 2016; Helletsgruber et al., 2017; Pang et al., 2021; Daniela et al., 2022).

The term “phyllosphere organisms” refers to organisms that are epiphytic or parasitic on the surface of plant leaves. Previous studies have shown that plants can synthesize or secrete various metabolites to affect their phyllosphere microbiome (Pang et al., 2021; Xu et al., 2021). There are significant differences in the concentration and composition of secondary metabolites, bacterial population size, and microbial community composition among different plants. Certain species of phyllosphere bacteria, such as *Methylobacterium extorquens* and the yeast *Candida boidinii*, can use VOCs such as methanol as growth substrates (Yurimoto and Sakai, 2009; Ochsner et al., 2015). This ability lends bacteria a selective advantage in the phyllosphere colonization of plants that emit large amounts of VOCs. Similarly, the terpenoids, phenylpropionic acids, and some aldehydes among the primary VOC components in plant leaves exhibit antibacterial activities that affect the colonization of microorganisms in the phyllosphere area (Devi et al., 2010; Gabriel and Zhang, 2020; Zhang et al., 2020). Burdon et al. (2018) found that in *Digitalis purpurea* pollinated by bees, volatile linalool in the nectar slowed the growth of bacteria in the phyllosphere environment.

Although many studies have provided insights into the community structure or dynamic basis of the phyllosphere microbiome, there are still many gaps in the study of the phyllosphere microbiome relative to the contribution of the rhizosphere microbiome to the secondary metabolites of the host plants. The study of the PSM–microbiome interaction has significance for crop cultivation, breeding, and other applied fields (Pang et al., 2021). In traditional crop breeding, breeders typically pay limited attention to the plant microbiome or PSM–microbiome interactions based on crop yield and other traits. In recent years, phyllosphere microorganisms have been found to regulate host stress resistance, and they are involved with VOC emissions (Matsumoto et al., 2021; Munir et al., 2022). Phyllosphere pathogenic microorganisms can alter plant VOC emissions by inducing plant defense responses or disrupting normal metabolism (Matsumoto et al., 2022). Toome et al. (2010) infected willow leaves with *Melampsora epitea* to study the influence of pathogens on the release pattern of VOCs from willow leaves. Compared with the control, the released amounts of sesquiterpenes and lipoxygenase products (LOX) in leaves infected with *Melampsora epitea* increased by 175 times and 10 times, respectively (Toome et al., 2010). However, the impact of commensal phyllosphere microbiota on the VOC emissions remains scarcely explored. Previously, the inhibition of their commensal phyllosphere microbiome while changing the composition and proportion of terpenes in the VOCs was found during the fumigation of *Sambucus nigra* with antibiotics that included the flowers and leaves (Peñuelas et al., 2014). This result suggests the potential link between the phyllosphere microbiome and VOCs in higher plants.

In this study, six different varieties of fingered citron are utilized to characterize their aroma compounds and phyllosphere bacteria communities. The aim of this study is to investigate the differences in the phyllosphere bacteria and VOCs of the different varieties of fingered citron and to explore the direct relationship between the phyllosphere bacteria and VOCs to provide a theoretical basis for cultivation and the breeding of fingered citron.

## Materials and Methods

### Sample Source and Collection Methods

Samples were collected at The Fingered Citron Base in Qiaolifang Village, Chisong Town, Jindong District, Jinhua, China. The following varieties of *Citrus medica* ‘Fingered’ were used for experiments: YangGuang (YG), CuiZhi (CZ), QingYiTongZi (QYTZ), XiuZhen (XZ), DaYeQingYi (DY), and KaiXin (KX). The sampling method was as follows. Five fruit trees were randomly selected from each variety, and nine leaves and two fruits were selected from each tree. The leaves were used for the determination of the phyllosphere bacteria, with a total of 45 leaves sampled from each variety and eight pooled samples in total, considering all six varieties. The fruits were used for the determination of the aroma compounds, with 10 randomly selected fruits sampled for each variety and six pooled samples in total.

### DNA Extraction

The total genomic DNA samples were extracted using the OMEGA Soil DNA Kit (M5635-02) (Omega Bio-Tek, Norcross, GA, United States) following the manufacturer’s instructions and stored at −20°C prior to further analysis. The quantity and quality of the extracted DNA were measured using a NanoDrop NC2000 spectrophotometer (Thermo Fisher Scientific, Waltham, MA, United States) and 1.0% agarose gel electrophoresis, respectively.

### 16S rRNA Gene Amplicon Sequencing

Polymerase chain reaction (PCR) amplification of the bacterial 16S rRNA genes in the V5–V7 region was performed using the forward primer 799F (5′-AACMGGATTAGATACCCKG-3′) and the reverse primer 1193R (5′-ACGTCATCCCCACCTTCC-3′). Sample-specific 7-bp barcodes were incorporated into the primers for multiplex sequencing. The PCR components contained 5 μL of the reaction buffer (5×), 5 μL of the GC buffer (5×), 2 μL of dNTP (2.5 mM), 1 μL of each forward primer (10 μM) and reverse primer (10 μM), 2 μL of the DNA template, 0.25 μL of the Q5 DNA polymerase, and 8.75 μL of ddH_2_O. Thermal cycling consisted of initial denaturation at 98°C for 2 min followed by 30 cycles consisting of denaturation at 98°C for 15 s, annealing at 55°C for 30 s, and extension at 72°C for 30 s, with a final extension of 5 min at 72°C. The PCR amplicons were purified with Vazyme VAHTSTM DNA Clean Beads (Vazyme, Nanjing, China) and quantified using the Quant-iT PicoGreen dsDNA Assay Kit (Invitrogen, Carlsbad, CA, United States). After the individual quantification step, the amplicons were pooled in equal amounts, and paired-end 2 × 250 bp sequencing was performed using the Illumina MiSeq platform with the MiSeq Reagent Kit v3 at Shanghai Personal Biotechnology Co., Ltd. (Shanghai, China).

### Sequence Analysis

Microbiome bioinformatics was performed with QIIME2 2019.4 (Bolyen et al., 2019) with slight modifications according to the official tutorials.^1^ Briefly, raw sequence data were demultiplexed using the demux plugin, followed by primer cutting with the cutadapt plugin (Martin, 2011). Sequences were then quality filtered, denoised, and merged, and chimeras were removed using the DADA2 plugin (Callahan et al., 2015). Non-singleton amplicon sequence variants (ASVs) were aligned with mafft (Katoh et al., 2002) and used to construct a phylogenetic with fasttree2 (Price et al., 2009). Alpha-diversity metrics [Chao1 (Chao, 1984), observed species, Shannon (Shannon, 1948), Simpson (Simpson, 1949)], and the beta diversity metrics [Bray–Curtis dissimilarity (Bray and Curtis, 1957)] were estimated using the diversity plugin with samples rarefied to 345 sequences per sample. The taxonomy was assigned to ASVs using the classify-sklearn naïve Bayes taxonomy classifier in the feature-classifier plugin (Bokulich et al., 2018) against the SILVA database (release 132).

### Bioinformatics and Statistical Analyses

The sequence data analyses were primarily performed using the QIIME2 and R packages (v3.2.0). The ASV-level alpha diversity indices, such as the Chao1 richness estimator, the Shannon diversity index, and the Simpson index, were calculated using the ASV table in QIIME2 and visualized as box plots. The beta diversity analysis was performed to investigate the structural variation in the microbial communities across samples using the Bray-Curtis metrics and nonmetric multidimensional scaling (NMDS) hierarchical clustering (Ramette, 2007). The taxonomic compositions and abundances were visualized using MEGAN (Huson et al., 2011) and GraPhlAn (Asnicar et al., 2015). The linear discriminant analysis effect size (LEfSe) was performed to detect differentially abundant taxa across groups using the default parameters (Segata et al., 2011).

### Analysis of the Aroma Compounds in Fingered Citron

Hs-spme-gc-ms was used to detect and analyze the VOC_*S*_ in six varieties of fingered citron fruits, and the relative odor activity value (ROAV) analysis was used to determine the key aromatic compounds affecting the aroma. An HP-5MS capillary column (30 m × 0.25 mm × 0.25 μm) was used. The injection temperature was 250°C, the carrier gas was helium gas, the flow rate was 1.0 mL/min, and the solvent delay was 5 min. Heating process: The initial temperature was kept at 40°C for 3 min, the temperature was increased from 3°C/min to 100°C, and the temperature was then increased to 230°C at 5°C/min for 20 min. For the electron ionization source, the electron energy was 70 eV, the ion source temperature was 230°C, the interface temperature was 250°C, the full scanning mode was used, and the mass scanning range was 20–3350 m/z.

### Qualitative and Quantitative Analysis of the Aroma Components

Qualitative: The volatile compounds in the fingered citron were identified using gas chromatography-mass spectrometry through the retention index (RI) and comparison with the NIST, and volatile compounds with positive and negative matching degrees greater than 800 were selected (Lee et al., 2018). The retention index was calculated using Equation 1:


(1) $$\mathrm{Retention}\,\mathrm{index}=100\times \text{n}+100\,\left({\text{t}}_{\text{a}}-t_{\text{n}}\right)/\left({\text{t}}_{\text{n}}+1-t_{\text{n}}\right)$$

where t_a_ is the retention time of the chromatographic peak a; and t_n_ and t_n + 1_ are the retention time of C_n_ and C_n + 1_, respectively.

Quantification: The relative contents of the volatile compounds in fingered citron were calculated according to the peak area normalization method:


(2) Relative⁢content⁢(‰)=M/N× 1000

where M is the peak area of the aroma substances of a single component; and N is the total peak area (Shui et al., 2019).

### Determination of Main Flavor Substances of Finger Citron by ROAV Method

The contribution of volatile flavor substances in fingered citron was evaluated by referring to the ROAV method, and then the primary flavor substances were determined (Gao et al., 2014). The components that contributed the most to the total flavor of fingered citron samples were determined as follows:

ROAV_max_ = 100, then other components (a):


(3) $${\text{ROAV}}_{\text{a}}\approx 100\times {\text{C}\%}_{\text{a}}/{\text{C}\%}_{\text{max}}\times {\text{T}}_{\text{max}}/{\text{T}}_{\text{a}}$$

where C%_a_ is the relative content of the volatile components (%); T_a_ is the sensory threshold of the volatile components (μg/kg); C%_max_ and T_max_ are the relative content (%) and sensory threshold (μg/kg), respectively, of the volatile components with the largest contribution to the overall aroma of the sample.

## Results and Analysis

### Diversity of the Phyllosphere Bacterial Community of Fingered Citron

**FIGURE 1:**
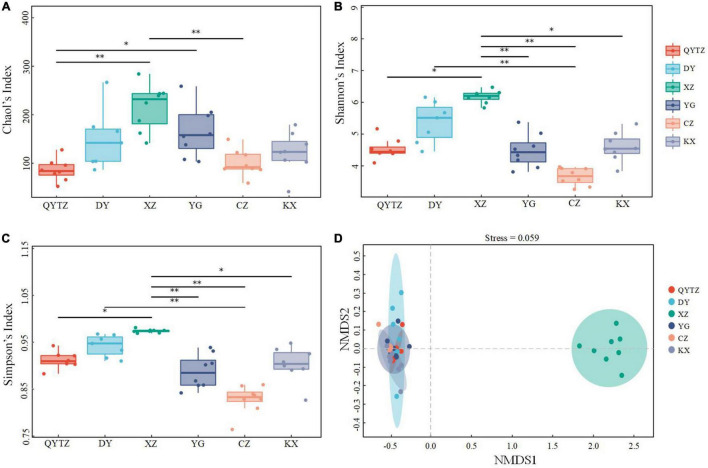
Microbial diversity of the phyllosphere bacteria of the different varieties. **(A)** Chao1 index; **(B)** Shannon index; **(C)** Simpson index; **(D)** NMDS analysis. *P < 0.05; **P < 0.01. QYTZ, QingYiTongZi; DY, DaYeQingYi; XZ, XiuZhen; YG, YangGuang; CZ, CuiZhi; KX, KaiXin.

According to the high-throughput sequencing of the 16S rDNA amplicon, this study conducted a diversity analysis of the phyllosphere microbial communities of different fingered citron cultivars (Figure 1). Only seven samples of DY were used for all analyses due to the poor DY-8 sequencing results. The α diversity Chao1, Shannon, and Simpson indices were utilized to measure the species richness and diversity in the group. Chao1 is the index of species richness, while the Shannon and Simpson indices are used to reflect the species diversity in samples (Jost, 2010). The Chao1 index showed that XZ had the highest microbial richness, and QYTZ had the lowest. The microbial richness of XZ was significantly different from those of QYTZ and CZ. The microbial richness of YG was significantly higher than that of QYTZ. There were no significant differences in the microbial richness among the other groups (Figure 1A). Both the Shannon and Simpson indices showed that XZ had the highest microbial diversity, while CZ had the lowest microbial diversity. The microbial diversity of XZ was significantly different from those of YG, CZ, KX, and QYTZ. The microbial diversity of DY was significantly higher than that of CZ. There was no significant difference in microbial diversity among other cultivars (Figures 1B,C).

The β diversity was used to compare the microbial community differences between varieties (Schloss et al., 2009). The NMDS analysis based on the Bray-Curtis distance was performed on the samples of different varieties of fingered citron. The results are shown in Figure 1D. The biological repeats of the same variety clustered together, indicating good repeatability. The difference between XZ and the other cultivars indicated that the microbial composition of XZ was significantly different from those of the other cultivars. QYTZ, DY, YG, CZ, and KX were clustered together, indicating similar microbial compositions among the five cultivars.

### The Phyllosphere Bacterial Community Structure of Fingered Citron

**FIGURE 2:**
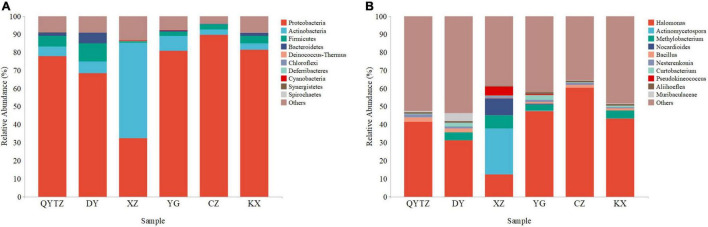
The relative abundances of the phyllosphere bacteria of the different varieties: **(A)** Relative abundances at the phylum level; **(B)** relative abundances at the genus level. QYTZ, QingYiTongZi; DY, DaYeQingYi; XZ, XiuZhen; YG, YangGuang; CZ, CuiZhi; KX, KaiXin.

The sequencing data showed that a total of 8,064,211 high-quality reads were obtained from six varieties of fingered citron. After splicing and quality control, a total of 2,760 ASVs were obtained. According to the ASV species annotation, there were 202 genera in 18 phyla. The top 10 phyla and genera with the highest relative abundances were selected for analysis. The top 10 phyla were as follows: Proteobacteria, Actinobacteria, Firmicutes, Bacteroidetes, Deinococcus-Thermus, Chloroflexi, Deferribacteres, Cyanobacteria, Synergistetes, and Spirochaetes (Figure 2A). Proteobacteria, Actinomycetes, and Firmicutes were present in all the samples with high relative abundances. Actinobacteria, Firmicutes, and Bacteroidetes were present in all six varieties of fingered citron. Proteobacteria reached a relative abundance of greater than 68% in all the varieties except XZ, and even 89.57% in CZ, while only 32.42% in XZ. Actinobacteria reached a relative abundance of 52.78% in XZ and less than 10% in the rest of the species. Firmicutes had the highest relative abundance in DY (10.05%), while it was only 0.88% in XZ. The relative abundances of Bacteroidetes in DY, KX, and QTYZ were 5.70, 2.02, and 1.79%, respectively, and less than 0.4% in the other three species. The relative abundances of the remaining phyla were low among the species.

At the genus level, *Halomonas* was present in all the samples and had a high relative abundance (Figure 2B). However, the relative abundance of *Halomonas* in XZ was significantly lower than that of the other varieties. The relative abundance of *Actinomycetospora* in XZ reached 25.28%, while in all the other species the relative abundance was less than 5%. The relative abundance of *Methylobacterium* in in QYTZ and CZ was 0.00%, and in XZ, KX, DY, and YG were 7.54, 4.36, 4.32, and 3.42%, respectively. The relative abundances of *Nocardioides* and *Pseudokineococcus* in XZ were significantly higher than those of the other species. The relative abundances of *Bacillus*, *Nesterenkonia*, and *Aliihoeflea* among all the samples were low, and the differences were not significant.

### Phyllosphere Bacterial Biomarkers in the Different Varieties

**FIGURE 3:**
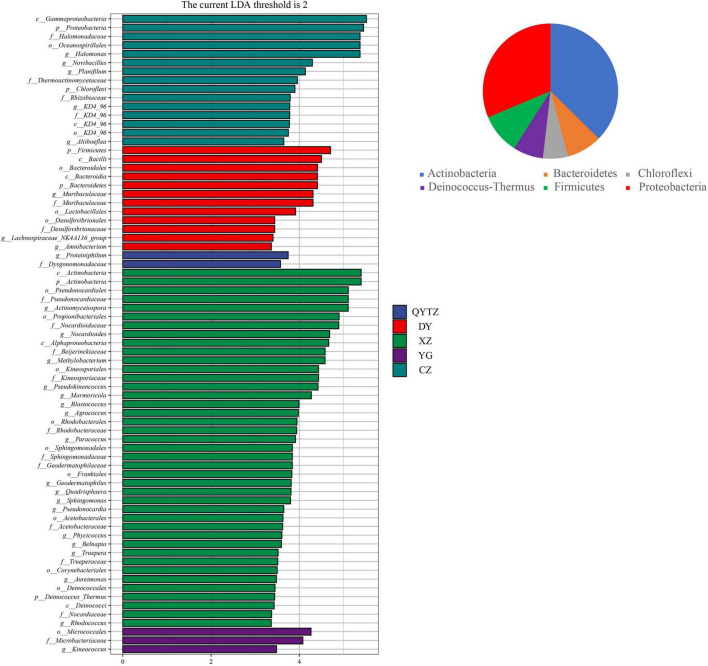
Linear discriminant analysis effect size (LEfSe) analysis of the phyllosphere bacteria of the different cultivars of fingered citron. The pie charts indicate the phylum in which the biomarker was located. QYTZ, QingYiTongZi; DY, DaYeQingYi; XZ, XiuZhen; YG, YangGuang; CZ, CuiZhi; KX, KaiXin.

The LEfSe analysis was used to analyze the enriched characteristic groups in the different varieties of fingered citron, and the LDA threshold was set to 2. The results are shown in Figure 3. XZ had the most biomarkers, involving 2 phyla, 3 classes, 9 orders, 10 families, and 16 genera, namely, *Actinomycetospora*, *Nocardiodies*, *Methylobacterium*, *Pseudokineococcus*, *Marmoriacola*, *Blastococcus*, *Agrococcus*, *Paracoccus*, *Quadrisphaera*, *Sphingomonas*, *Pseudonocardia*, *Phycicoccus*, *Belnapia*, *Truepera*, *Aureimonas*, and *Rhodococcus.* There were 15 biomarkers in CZ, involving two phyla, two classes, two orders, four families, and five genera. At the genus level, the relative abundances of *Halomonas*, *Nocardioides*, *Planifilum*, *KD4-96*, and *Aliihoeflea* in CZ were significantly higher than in the other varieties, and these high abundances were biomarkers of CZ. The biomarkers in DY involved two phyla, two classes, three orders, two families, and three genera. The genera included *Muribaculaceae*, *Lachnospiraceae_NK4A136_group*, and *Amnibacterium*. The biomarkers of QYTZ involved one family and one genus (Dysgonomonadaceae and *Proteiniphilum*, respectively). The markers of YG involved one order, one family, and one genus. The relative abundance of *Kineococcus* in YG was significantly higher than that in the other varieties. KX had no biomarker. All biomarkers were distributed in six phyla, except *Chloroflexi*, which was in the top five relative abundances of each variety.

### Structure and Diversity of Volatile and Aromatic Components of Fingered Citron

**FIGURE 4:**
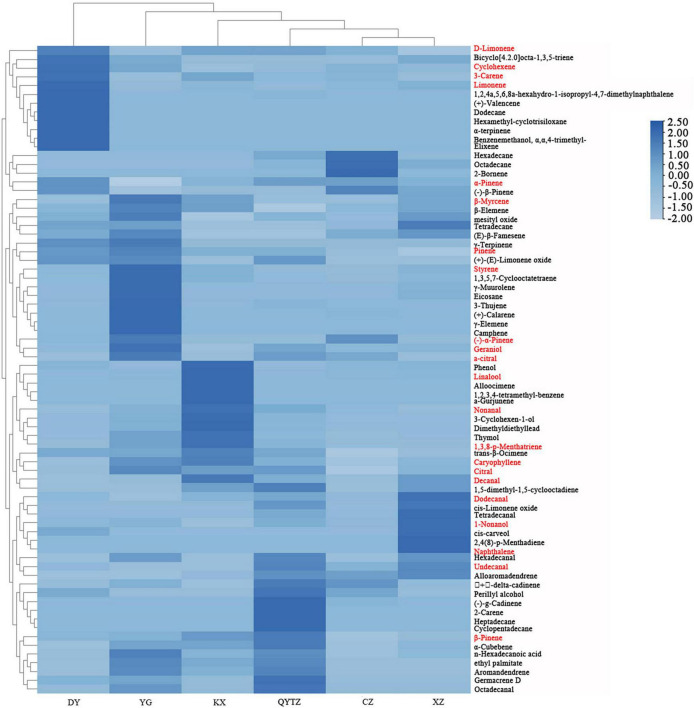
Heat map of the volatile substances in the six fingered citron varieties. The red color means an ROAV ≥ 1. QYTZ, QingYiTongZi; DY, DaYeQingYi; XZ, XiuZhen; YG, YangGuang; CZ, CuiZhi; KX, KaiXin.

The names and relative contents of the volatile flavor compounds in the different fingered citron varieties are displayed in Supplementary Table 1. A total of 76 types of volatile flavor compounds were detected in six varieties. The quantity and relative contents of the volatile flavor compounds in the different fingered citron varieties differed to some extent. As shown in Figure 4, the specific volatile substances in the variety DY included dodecane, hexamethyl-cyclotrisiloxane, benzenemethanol, α,α,4- trimethyl-, α-terpinene, elixene, and (+)-valencene. The characteristic volatile compounds of QY were 2-carene, cyclopentadecane, and heptadecane. The unique volatiles of CZ included 2-bornene. The KX characteristic volatile substances were alloocimene, 1,2,3,4-tetramethyl-benzene, and a-gurjunene. The unique volatile substances in XZ were 2,4(8)-p-menthadiene and naphthalene. The unique volatile substances in YG were γ-elemene and camphene. According to the cluster analysis of the volatile compounds in the heat map, the volatile components of CZ and XZ were the most similar and could be roughly divided into one category, while some components exhibited content differences. The volatiles of the other four varieties could be roughly divided into one category, and the volatile components of DY were the most different from those of the other five varieties.

A variety of volatile flavor compounds were detected in the different fingered citron cultivars, but only a subset of them contributed to the overall fruit aroma. The remaining ones only played a modifiable and synergistic role in the presentation of the overall flavor of fingered citron. The contributions of VOCs to the characteristic aroma of fingered citron were determined by their relative contents and the aroma threshold, which is the minimum smellable value of odor. Hence, the relative content of VOCs did not explain its contribution to the flavor of fingered citron. Therefore, the ROAV was analyzed based on the threshold values of each VOC. Substances with ROAV ≥ 1 contributed significantly to the aroma of samples and were considered to be key aroma compounds. The larger the ROAV value was, the greater the contribution to the total flavor of the sample was.

There were 22 key aroma compounds, that contributed significantly to the aroma of fingered citron, varying considerably between varieties (Table 1). DY had five key aroma compounds, with pinene having a greater impact on its overall flavor. QYTZ had six species, with pinene and geraniol having a greater impact on its overall flavor. CZ had four species, with pinene and linalool having a greater impact on its overall flavor. KX had three species, with linalool having a greater impact on its overall flavor. XZ had five types, with linalool and geraniol having a greater impact on its overall flavor. YG had 14 species, with pinene and linalool having a greater impact on its overall flavor.

**TABLE 1:** The aroma compounds of ROAV ≥ 1 in the different varieties of fingered citron and their thresholds and odor quality.

No.	Compounds	Threshold (μg/kg)	ROAV					
			
			DY	QY	CZ	KX	XZ	YG
A1	Styrene	0.0036	–	–	–	–	1.178	2.158
A2	Pinene	0.0022	921.6	401.9	465.6	396.4	280	1134
A3	α-Pinene	0.014	75.38	42.27	94.27	33.31	161.1	25.28
A4	β-Pinene	0.14	10.22	10.28	9.671	8.329	24.64	11.08
A5	β-Myrcene	0.0012	–	–	–	11.78	48.3	35.76
A6	D-Limonene	0.034	253.4	100	173.8	100	–	47.93
A7	Limonene	0.2	100	9.922	32.3	15.28	100	16.95
A8	(-)-α-Pinene	0.1	13.23	–	100	–	–	100
A9	Cyclohexene	0.2	14.21	1.073	5.65	–	7.083	6.614
A10	Caryophyllene	0.064	5.851	4.689	6.961	6.425	19.47	9.626
A11	3-Carene	0.77	6.193	–	1.964	1.663	–	–
A12	1,3,8-p-Menthatriene	0.015	–	–	–	1.238	–	–
A13	Nonanal	0.0011	–	11.29	7.116	29.15	–	14
A14	Decanal	0.003	1.768	5.705	3.724	12.37	37.78	3.514
A15	a-Citral	0.032	–	4.173	7.352	–	–	11.31
A16	Undecanal	0.0125	–	1.858	1.616	–	8.142	–
A17	Citral	0.17	–	1.007	–	–	2.691	2.023
A18	Dodecanal	0.01	–	–	–	–	6.798	–
A19	Linalool	0.00022	242.6	165.5	446.5	1174	998.3	320.3
A20	Geraniol	0.0011	177.9	282	271.6	0	712.3	955.9
A21	1-Nonanol	0.0455	–	–	–	–	1.347	–
A22	Naphthalene	0.006	–	–	–	–	2.441	–

### Correlation Between Fruit Volatiles and Bacterial Microbiota

**FIGURE 5:**
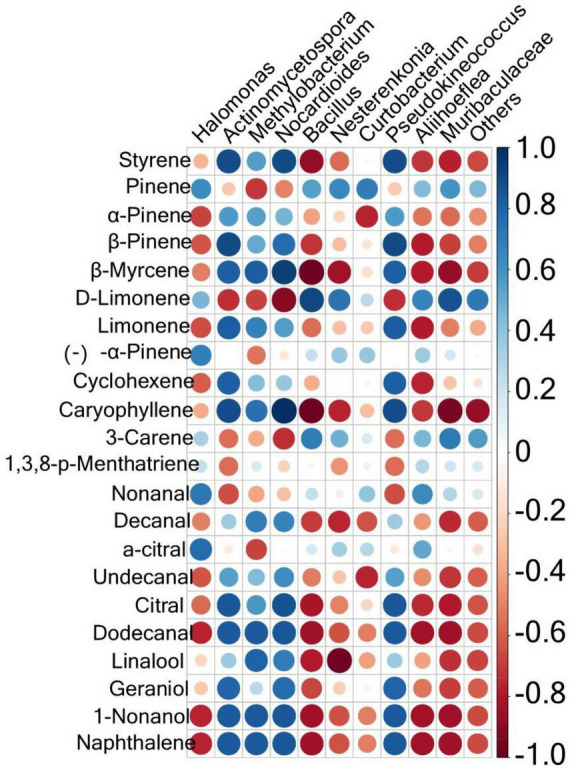
Correlation analysis of the phyllosphere bacterial communities and the aroma compounds in the fingered citron. The circle size is positively correlated with the correlation coefficient.

To explore the correlation between microbial components and volatile substances, a Spearman correlation analysis was conducted between the microorganisms with the top 10 relative abundances at the genus level and 22 key aroma substances with ROAV ≥ 1. The results are shown in Figure 5. Results with *P* < 0.05 and R > 0.6 were further screened, as shown in Supplementary Table 2. There were 55 positive correlations and 60 negative correlations between microbes and volatile compounds. The most volatile compounds were positively correlated with *Nocardioides*. These included styrene, β-pinene, β-myrcene, caryophyllene, decanal, undecanal, citral, dodecanal, linalool, geraniol, 1-nonanol, and naphthalene. *Curtobacterium* was positively correlated with pinene only. Twelve volatile species were negatively correlated with *Muribaculaceae*: styrene, β-pinene, β-myrcene, caryophyllene, decanal, undecanal, citral, dodecanal, linalool, geraniol, 1-nonanol, and naphthalene. *Actinomycetospora* and *Pseudokineococcus* were both negatively correlated with only two volatile compounds (D-limonene and nonanal). *Nocardioides* was positively correlated with D-limonene and 3-carene.

## Discussion

### Similarities and Differences Between Different Fingered Citron Cultivars

Plant host-associated microbial communities may be formed by a variety of environmental and host-related factors, including geographic location, plant phenotypes and genotypes, soil nutrients, and seasonal effects (Lindow and Brandl, 2003; Vorholt, 2012). In this study, it was found that the phyllosphere bacteria of fingered citron were primarily Proteobacteria, Actinobacteria, Firmicutes, and Bacteroidetes. The first three types of microorganisms had high relative abundances in all of the fingered citron varieties, while Bacteroidetes only had high relative abundance in some samples of *Citrus medica* ‘Fingered’ (DaYeQingYi) and *Citrus medica* ‘Fingered’ (KaiXin) (Figure 2A). Proteobacteria, Actinobacteria, Firmicutes, and Bacteroidetes were the primary phyllosphere bacterial communities (Rastogi et al., 2012; Trivedi et al., 2020). Proteobacteria have a variety of functions, such as methyl nutrition, nitrification, and nitrogen fixation, in the phyllosphere bacterial community (Michael et al., 2008; Atamna-Ismaeel et al., 2012), and reports have indicated that Proteobacteria make up a high proportion of the phyllosphere bacteria of studied plants (Trivedi et al., 2020). Actinomycetes and Firmicutes are closely related to plant resistance and nitrogen fixation (Sasikumar et al., 2013). Bacteroidetes are considered a specific phylum in that biosphere that degrades complex organic matter (Huang et al., 2020). The differences in the secondary metabolites between the different plants of the same variety may be the reason for the large variation within the group.

There are adaptive matches between phyllosphere bacteria and their plant hosts (Lajoie et al., 2020; Whipps et al., 2008). As shown in Figure 2B, there were obvious differences between the phyllosphere bacteria of XZ and other varieties. Actinomycetes had the highest relative abundance in XZ, followed by *Halomonas*. *Halomonas* was a common genus among all samples, and it was the genus with the highest relative content in all the cultivars except for XZ. In addition, *Bacillus*, *Nesterenkonia*, and *Aliihoeflea* were found in all the cultivars, but their relative abundances were low.

The community structure and diversity of phyllosphere bacteria are influenced by cultivation conditions, plant species and genotypes, leaf age, and other factors (Zhiwei, 1995; Hirano and Upper, 2000; Finkel et al., 2012). XZ had the highest microbial richness and diversity, with biomarkers of 17 genera. The NMDS analysis clearly distinguished XZ from the other varieties, indicating that variety was one of the conditions affecting the phyllosphere bacterial community structure.

### Similarities and Differences in the VOCs Among the Different Varieties of Fingered Citron

Aroma is an important index used to measure fruit quality, and VOCs vary between varieties. A total of 76 VOCs were detected in six different varieties of fingered citron (Supplementary Table 1). The results were consistent with those obtained using steam distillation and SPME, which showed that alkenes, alcohols, and aldehydes were more predominant in the different species of fingered citron (Xia et al., 2018). α-cubebene, caryophyllene, and (+)-delta-cadinene were identified in all of the varieties.

Fingered citron fruit extract has anti-cancer, anti-oxidant, and anti-free radical effects and is a raw material for anti-bacterial agents, pharmaceuticals, and food additives (Wang et al., 2020). Volatile oils, also known as aromatic oils, and essential oils are an important part of the fingered citron extract. In this study, it was found that the fruits of different varieties of fingered citron possess their own specific volatiles. VOCs of the fingered citron are not only related to variety, but also are constantly changing as the fruit develops and matures. Aldehydes, esters, and acid compounds were the primary volatile substances in the fingered citrons harvested in Guangdong Province in June and July, while the primary volatile substances in the fingered citrons harvested in August and September included alcohols, aldehydes, esters, alkanes, and phenols at low levels (Tang et al., 2021). Therefore, when making further processed products of fingered citron, attention should be paid to the choice of raw material, especially the variety and the time of harvesting, so that more of the active substance can be obtained.

### The Relationship Between VOCs and the Bacterial Microbiota

It has been demonstrated that microbial community structure affects fruit volatiles. For example, 2,5-dimethyl-4-hydroxy-2H-furan-3-one from strawberries is associated with *Methylobacterium* (Nasopoulou et al., 2014). Raspberries contain *Bacillus*, *Lactobacillus*, *Bacteroides*, and other bacteria that can increase the volatile substances in raspberries (Daniela et al., 2022). These microbes not only produce volatile compounds that enhance the fruit’s flavor but also enhance its ability to defend against pests and pathogens (Daranas et al., 2019). *Bacillus* stimulates plant growth, and recent research has found that *Bacillus subtilis* in citrus trees can effectively resist the Huanglongbing pathogen (Daivasikamani, 2009; Yu et al., 2011; Munir et al., 2022). In strawberry and apples, lactic acid bacteria can be used as a biological control agent for disease (Trias et al., 2009; Tsuda et al., 2013; Zhou et al., 2017). In this study, it was found that *Halomonas* were significantly positively correlated with pinene, α-pinene, nonanal, and a-citral, among which, pinene and α-pinene were key volatiles in all of the fingered citron varieties. Actinomycetes were significantly positively correlated with styrene, β-pinene, β-myrcene, limonene, cyclohexene, caryophyllene, citral, dodecanal, geraniol, 1-nonol, and naphthalene, and the contents of these volatile compounds were higher in XZ. However, whether these volatiles are produced by bacterial enrichment requires further study. It was found that *Bacillus* could produce nonanol in raspberries (Daniela et al., 2022), but the correlation between *Bacillus* and nonanol was not significant in this study. It requires further investigation as to whether this phenomenon is caused by the difference in host plants or the weak correlation due to the small sample size.

In addition to the volatiles produced by some bacteria that increase fruit quality, the volatiles produced by fruits also cause changes in the bacterial community structure. Citrus plants produce a large number of volatile substances that affect the microbial community composition of the phyllosphere. The volatile oil of fingered citron had significant inhibitory effects on the growth of gram-positive bacteria, gram-negative bacteria, and yeast, especially on *Bacillus subtilis* and *Escherichia coli*, which reduced the diversity of microorganisms in fingered citron (Guo et al., 2009; Gao et al., 2020). In this study, it was found that there was a negative correlation between *Bacillus* and 11 key volatile compounds, and the relative abundance of *Bacillus* was low in the leaves, and this might have been caused by the bacteriostatic effect of the volatile oil of the fingered citron. Specific volatile substances with inhibitory effects on *Bacillus* need to be identified in further experiments.

Correlations between bacteria and volatiles may indicate direct bacterial production or the utilization of volatiles, the enhanced release of volatiles from plant tissues, the selective effects of VOCs on specific bacterial groups, or even indirect non-causal relationships (Daniela et al., 2022). The interaction between the bacterial community and volatile compounds, the environment, host plants, and other factors may have resulted in the different aroma characteristics of the different varieties.

## Conclusion and Prospects

In this study, the HS-SPME and ROAV combined analysis was used to determine the key aroma compounds of different fingered citron varieties. The contribution of bacterial microflora to the aroma of fingered citron fruits was investigated. The correlation analysis highlighted several significant associations of bacterial genera and aroma emissions that may explain the complexity of the analysis of the bacterial and metabolic interactions. This study revealed the characteristics of the core microbiota in the phyllosphere area of the fingered citron for the first time. Based on the results of this study, the naturally parasitic bacteria in the phyllosphere area of fingered citron may be associated with the production and emission of aromatic substances. In the future, to further determine the association between phyllosphere bacteria and VOCs of fingered citron, high-throughput sequencing will be used to further explore the composition of the phyllosphere bacterial community of fingered citron at the species level, and bacteria (such as Bacillus and Halomonas) with significant associations with VOCs on fingered citron leaves will be extracted, isolated, and cultured. The cultured bacteria will be inoculated onto the leaves of the fingered citron, and the changes in the VOCs of the fingered citron fruits will be measured. Hence, their correlations will be determined.

## Data Availability Statement

The datasets presented in this study can be found in online repositories. The names of the repository/repositories and accession number(s) can be found below: https://ngdc.cncb.ac.cn/gsa/, PRJCA009805.

## Author Contributions

YW and PS designed the study. JW, CC, and YW conducted the experiment and collected the samples. JS, JW, and YW analyzed the data and wrote the manuscript. JS and YW critically reviewed the manuscript. All authors approved the final manuscript.

## Conflict of Interest

The authors declare that the research was conducted in the absence of any commercial or financial relationships that could be construed as a potential conflict of interest.

## Publisher’s Note

All claims expressed in this article are solely those of the authors and do not necessarily represent those of their affiliated organizations, or those of the publisher, the editors and the reviewers. Any product that may be evaluated in this article, or claim that may be made by its manufacturer, is not guaranteed or endorsed by the publisher.

## Footnotes

1
https://docs.qiime2.org/2019.4/tutorials/
