# Supplementary material for: Community Structure of Phyllosphere Bacteria in Different Cultivars of Fingered Citron (Citrus medica ‘Fingered’) and Their Correlations With Fragrance
Source: Front Plant Sci. 2022 Jul 15;13:936252. doi: 10.3389/fpls.2022.936252 (PMC9335054; doi:10.3389/fpls.2022.936252)
Supplement: Supplementary file 1 [file Data_Sheet_1.docx]

Supplementary Table 1 Relative content of volatile compounds in different fingered citron

| NO. | Compounds | CAS | RI | | Content (‰) | | | | | |
| --- | --- | --- | --- | --- | --- | --- | --- | --- | --- | --- |
|  |  |  | Calculate | Reference | DY | QY | CZ | KX | XZ | YG |
| 1 | Bicyclo[4.2.0]octa-1,3,5-triene | 694-87-1 | 869 | —— | 0.008 | —— | —— | —— | 0.003 | 0.003 |
| 2 | Styrene | 100-42-5 | 890 | 890 | 0.018 | 0.005 | —— | 0.042 | 0.033 | 0.168 |
| 3 | 1,3,5,7-Cyclooctatetraene | 629-20-9 | 891 | —— | 0.002 | 0.001 | —— | 0.008 | 0.004 | 0.037 |
| 4 | Pinene | 7785-70-8 | 897 | —— | 43.95 | 31.82 | 16.23 | 31.65 | 4.793 | 53.96 |
| 5 | α-Pinene | 80-56-8 | 931 | 917 | 22.88 | 21.3 | 20.91 | 16.93 | 17.55 | 7.654 |
| 6 | (-)-β-Pinene | 18172-67-3 | 933 | —— | 13.17 | 2.856 | 15.9 | 2.959 | 9.553 | —— |
| 7 | β-Pinene | 127-91-3 | 885 | 964 | 31.02 | 51.78 | 21.45 | 42.33 | 26.84 | 33.54 |
| 8 | β-Myrcene | 123-35-3 | 974 | 981 | —— | —— | —— | 0.513 | 0.451 | 0.928 |
| 9 | γ-Terpinene | 99-85-4 | 975 | 1064 | 9.148 | —— | —— | —— | —— | 12.43 |
| 10 | D-Limonene | 5989-27-5 | 1000 | —— | 186.7 | 122.4 | 93.64 | 123.4 | —— | 35.24 |
| 11 | Limonene | 138-86-3 | 1024 | 1030 | 433.6 | 71.42 | 102.3 | 110.9 | 155.6 | 73.33 |
| 12 | (-)-α-Pinene | 7785-26-4 | 1080 | 936 | 28.68 | —— | 158.4 | —— | —— | 216.3 |
| 13 | Cyclohexene | 586-62-9 | 1092 | 1097 | 61.59 | 7.724 | 17.9 | 1.939 | 11.02 | 28.61 |
| 14 | trans-β-Ocimene | 7216-56-0 | 1116 | 1131 | 2.746 | 2.829 | 1.064 | 3.839 | 1.717 | 2.84 |
| 15 | (+)-(E)-Limonene oxide | 6909-30-4 | 1140 | —— | 0.259 | 0.248 | —— | —— | —— | 0.304 |
| 16 | Alloaromadendrene | 25246-27-9 | 1311 | 1457 | —— | 0.105 | 0.083 | 0.024 | 0.114 | —— |
| 17 | α-Cubebene | 17699-14-8 | 1334 | 1354 | 0.205 | 0.902 | 0.183 | 0.587 | 0.362 | 0.584 |
| 18 | Germacrene D | 23986-74-5 | 1353 | 1480 | 3.46 | 12.93 | 0.116 | 0.229 | —— | 5.746 |
| 19 | β-Elemene | 515-13-9 | 1392 | 1394 | 0.151 | —— | 0.133 | 0.243 | 0.22 | 0.333 |
| 20 | Caryophyllene | 87-44-5 | 1404 | 1417 | 8.117 | 10.8 | 7.057 | 14.93 | 9.698 | 13.32 |
| 21 | γ-Muurolene | 30021-74-0 | 1428 | 1474 | —— | —— | —— | —— | 0.11 | 0.66 |
| 22 | (E)-β-Famesene | 18794-84-8 | 1455 | 1459 | 0.447 | —— | 0.419 | —— | 0.619 | 0.748 |
| 23 | (+)-Calarene | 17334-55-3 | 1461 | 1440 | —— | —— | 0.03 | —— | —— | 0.455 |
| 24 | (+)-delta-cadinene | 483-76-1 | 1600 | 1519 | 0.705 | 1.774 | 1.46 | 0.622 | 0.779 | 1.062 |
| 25 | 3-Carene | 13466-78-9 | 1083 | 1005 | 103.4 | 14.12 | 23.96 | 46.46 | —— | —— |
| 26 | α-terpinene | 99-86-5 | 1244 | 1017 | 0.03 | —— | —— | —— | —— | —— |
| 27 | 3-Thujene | 2867/5/2 | 925 | 911 | —— | 0.77 | 0.129 | —— | —— | 7.771 |
| 28 | 1,5-dimethyl-1,5-cyclooctadiene | 3760-14-3 | 1044 | —— | —— | 256.4 | —— | 159.7 | 174 | —— |
| 29 | 2-Carene | 554-61-0 | 1051 | 1001 | —— | 25.05 | —— | —— | —— | —— |
| 30 | 1,3,8-p-Menthatriene | 18368-95-1 | 1127 | 1118.7 | —— | 0.125 | 0.002 | 0.674 | —— | 0.309 |
| 31 | Aromandendrene | 489-39-4 | 1382 | 1440 | —— | 0.046 | —— | 0.019 | —— | 0.042 |
| 32 | γ-Elemene | 29873-99-2 | 1237 | —— | —— | —— | —— | —— | —— | 0.053 |
| 33 | 2-Bornene | 464-17-5 | 1248 | —— | —— | —— | 0.223 | —— | —— | —— |
| 34 | Alloocimene | 673-84-7 | 1111 | —— | —— | —— | —— | 11.68 | —— | —— |
| 35 | a-Gurjunene | 489-40-7 | 1409 | —— | —— | —— | —— | 0.032 | —— | —— |
| 36 | 2,4(8)-p-Menthadiene | 586-63-0 | 1244 | 1085 | —— | —— | —— | —— | 0.334 | —— |
| 37 | Camphene | 3387-41-5 | 1000 | 961 | —— | —— | —— | —— | —— | 1.125 |
| 38 | (+)-Valencene | 4630/7/3 | 1492 | 1491 | 0.018 | —— | —— | —— | —— | —— |
| 39 | Heptadecane | 629-78-7 | 1699 | —— | —— | 0.008 | —— | —— | —— | —— |
| 40 | cis-Limonene oxide | 13837-75-7 | 1138 | 1131 | —— | 0.071 | —— | —— | 0.118 | —— |
| 41 | Tetradecane | 629-59-4 | 1394 | —— | 0.056 | —— | 0.019 | —— | 0.099 | 0.062 |
| 42 | Hexadecane | 544-76-3 | 1800 | —— | —— | 0.023 | 0.069 | —— | —— | —— |
| 43 | Octadecane | 593-45-3 | 1817 | —— | —— | 0.003 | 0.021 | —— | 0.006 | —— |
| 44 | Dodecane | 112-40-3 | 2000 | —— | 0.044 | —— | —— | —— | —— | —— |
| 45 | Hexamethyl-cyclotrisiloxane | 541-05-9 | 830 | —— | 0.072 | —— | —— | —— | —— | —— |
| 46 | Cyclododecane | 294-62-2 | 1576 | —— | —— | —— | —— | —— | —— | —— |
| 47 | Cyclopentadecane | 295-48-7 | 1712 | —— | —— | 0.002 | —— | —— | —— | —— |
| 48 | Hexadecyl-oxirane | 7390-81-0 | 1920 | —— | —— | —— | —— | —— | —— | —— |
| 49 | Elixene | 3242/8/8 | 1341 | —— | 0.124 | —— | —— | —— | —— | —— |
| 50 | Eicosane | 112-95-8 | 1900 | —— | —— | —— | —— | —— | 0.003 | 0.016 |
| 51 | Nonanal | 124-19-6 | 1114 | 1089 | —— | 0.447 | 0.124 | 1.164 | —— | 0.333 |
| 52 | Decanal | 112-31-2 | 1181 | 1200 | 0.115 | 0.616 | 0.177 | 1.347 | 0.882 | 0.228 |
| 53 | a-Citral | 141-27-5 | 1269 | 1269 | —— | 4.806 | 3.727 | —— | —— | 7.829 |
| 54 | Undecanal | 112-44-7 | 1301 | 1308 | 0.168 | 0.836 | 0.32 | —— | 0.792 | —— |
| 55 | Octadecanal | 638-66-4 | 1995 | 2024 | —— | 0.025 | —— | —— | —— | 0.015 |
| 56 | Hexadecanal | 629-80-1 | 1715 | 1811 | —— | 0.048 | —— | —— | 0.04 | 0.035 |
| 57 | Tetradecanal | 124-25-4 | 1614 | 1614 | —— | 0.056 | —— | —— | 0.171 | —— |
| 58 | Citral | 5392-40-5 | 1177 | —— | 2.128 | 6.164 | —— | 5.901 | 3.56 | 7.436 |
| 59 | Dodecanal | 112-54-9 | 1413 | 1412 | 0.108 | 0.248 | 0.093 | 0.157 | 0.529 | —— |
| 60 | Linalool | 78-70-6 | 1098 | 1104 | 1.157 | 1.31 | 1.556 | 9.375 | 1.709 | 1.524 |
| 61 | 3-Cyclohexen-1-ol | 20126-76-5 | 1144 | 1175 | 1.577 | 3.803 | 1.384 | 17.558 | 1.255 | 5.32 |
| 62 | Geraniol | 106-24-1 | 1210 | 1249 | 4.242 | 11.16 | 4.733 | —— | 6.097 | 22.74 |
| 63 | Perillyl alcohol | 536-59-4 | 1296 | 1302.7 | 0.029 | 0.074 | 0.034 | —— | —— | —— |
| 64 | 1-Nonanol | 143-08-8 | 1179 | 1186 | 0.085 | 0.176 | 0.082 | —— | 0.477 | 0.128 |
| 65 | Benzenemethanol, α,α,4-trimethyl- | 1197-01-9 | 1191 | 1188 | 0.034 | —— | —— | —— | —— | —— |
| 66 | cis-carveol | 1197-06-4 | 1225 | 1220 | 0.2 | —— | —— | —— | 0.602 | —— |
| 67 | 1,2,3,4-tetramethyl-benzene | 488-23-3 | 1122 | —— | —— | —— | —— | 0.025 | —— | —— |
| 68 | [mesityl oxide](https://mip.chem960.com/cas/141797/) | 141-79-7 | 800 | 798 | 2.662 | 2.509 | 2.227 | 1.756 | 3.085 | 3.576 |
| 69 | 1,2,4a,5,6,8a-hexahydro-1-isopropyl-4,7-dimethylnaphthalene | 483-75-0 | 1482 | 1452 | 0.588 | 0.043 | —— | —— | —— | —— |
| 70 | (-)-g-Cadinene | 39029-41-9 | 1596.23 | 1511 | —— | 1.297 | 0.105 | —— | —— | —— |
| 71 | Naphthalene | 91-20-3 | 1185 | 1178 | —— | —— | —— | —— | 0.114 | —— |
| 72 | ethyl palmitate | 628-97-7 | 2000 | 1993 | —— | 0.013 | —— | 0.007 | —— | 0.013 |
| 73 | Phenol | 499-75-2 | 1283 | 1317 | 0.098 | —— | 0.065 | 0.705 | 0.105 | —— |
| 74 | Thymol | 89-83-8 | 1291 | 1297 | 0.154 | 0.251 | 0.043 | 1.675 | 0.15 | 0.818 |
| 75 | n-Hexadecanoic acid | 1957/10/3 | 1959 | 1964 | 0.005 | 0.045 | —— | 0.019 | 0.012 | 0.053 |
| 76 | Dimethyldiethyllead | 1762-27-2 | 1172 | —— | —— | 0.047 | —— | 0.457 | —— | 0.101 |
|  |  |  |  |  |  |  |  |  |  |  |

Supplementary Table 2 Correlation analysis of the interleaf bacterial community and aroma compounds of fingered citron

| Bacterial Genus | Compounds | P | R |
| --- | --- | --- | --- |
| Actinomycetospora | Styrene | 0.00 | 0.88 |
|  | β-Pinene | 0.00 | 0.90 |
|  | β-Myrcene | 0.00 | 0.81 |
|  | Limonene | 0.00 | 0.83 |
|  | Cyclohexene | 0.00 | 0.81 |
|  | Caryophyllene | 0.00 | 0.88 |
|  | Citral | 0.00 | 0.85 |
|  | Dodecanal | 0.00 | 0.83 |
|  | Geraniol | 0.00 | 0.80 |
|  | 1-Nonanol | 0.00 | 0.83 |
|  | Naphthalene | 0.00 | 0.83 |
|  | D-Limonene | 0.00 | -0.73 |
|  | Nonanal | 0.00 | -0.65 |
| Aliihoeflea | D-Limonene | 0.00 | 0.67 |
|  | Nonanal | 0.00 | 0.63 |
|  | Styrene | 0.00 | -0.71 |
|  | β-Pinene | 0.00 | -0.78 |
|  | β-Myrcene | 0.00 | -0.78 |
|  | Limonene | 0.00 | -0.80 |
|  | Cyclohexene | 0.00 | -0.76 |
|  | Caryophyllene | 0.00 | -0.71 |
|  | Citral | 0.00 | -0.74 |
|  | Dodecanal | 0.00 | -0.83 |
|  | 1-Nonanol | 0.00 | -0.83 |
|  | Naphthalene | 0.00 | -0.83 |
| Bacillus | D-Limonene | 0.00 | 0.91 |
|  | 3-Carene | 0.00 | 0.69 |
|  | Styrene | 0.00 | -0.88 |
|  | β-Pinene | 0.00 | -0.71 |
|  | β-Myrcene | 0.00 | -0.98 |
|  | Caryophyllene | 0.00 | -0.98 |
|  | Decanal | 0.00 | -0.69 |
|  | Citral | 0.00 | -0.82 |
|  | Dodecanal | 0.00 | -0.85 |
|  | Linalool | 0.00 | -0.78 |
|  | Geraniol | 0.00 | -0.66 |
|  | 1-Nonanol | 0.00 | -0.85 |
|  | Naphthalene | 0.00 | -0.85 |
| Curtobacterium | Pinene | 0.00 | 0.69 |
|  | α-Pinene | 0.00 | -0.77 |
|  | Decanal | 0.00 | -0.63 |
|  | Undecanal | 0.00 | -0.77 |
| Halomonas | Pinene | 0.00 | 0.62 |
|  | (-) -α-Pinene | 0.00 | 0.68 |
|  | Nonanal | 0.00 | 0.72 |
|  | a-Citral | 0.00 | 0.76 |
|  | α-Pinene | 0.00 | -0.68 |
|  | β-Pinene | 0.00 | -0.62 |
|  | Limonene | 0.00 | -0.64 |
|  | Cyclohexene | 0.00 | -0.61 |
|  | Undecanal | 0.00 | -0.62 |
|  | Dodecanal | 0.00 | -0.77 |
|  | 1-Nonanol | 0.00 | -0.77 |
|  | Naphthalene | 0.00 | -0.77 |
| Methylobacterium | β-Myrcene | 0.00 | 0.83 |
|  | Limonene | 0.00 | 0.67 |
|  | Caryophyllene | 0.00 | 0.75 |
|  | Decanal | 0.00 | 0.70 |
|  | Dodecanal | 0.00 | 0.83 |
|  | Linalool | 0.00 | 0.80 |
|  | 1-Nonanol | 0.00 | 0.83 |
|  | Naphthalene | 0.00 | 0.83 |
|  | Pinene | 0.00 | -0.71 |
|  | D-Limonene | 0.00 | -0.67 |
|  | a-Citral | 0.00 | -0.68 |
| Muribaculaceae | D-Limonene | 0.00 | 0.86 |
|  | 3-Carene | 0.00 | 0.70 |
|  | Styrene | 0.00 | -0.78 |
|  | β-Pinene | 0.00 | -0.69 |
|  | β-Myrcene | 0.00 | -0.86 |
|  | Caryophyllene | 0.00 | -0.95 |
|  | Decanal | 0.00 | -0.74 |
|  | Undecanal | 0.00 | -0.72 |
|  | Citral | 0.00 | -0.80 |
|  | Dodecanal | 0.00 | -0.84 |
|  | Linalool | 0.00 | -0.72 |
|  | Geraniol | 0.00 | -0.69 |
|  | 1-Nonanol | 0.00 | -0.84 |
|  | Naphthalene | 0.00 | -0.84 |
| Nesterenkonia | Pinene | 0.00 | 0.65 |
|  | D-Limonene | 0.00 | 0.73 |
|  | β-Myrcene | 0.00 | -0.84 |
|  | Caryophyllene | 0.00 | -0.77 |
|  | Decanal | 0.00 | -0.75 |
|  | Dodecanal | 0.00 | -0.64 |
|  | Linalool | 0.00 | -0.96 |
|  | 1-Nonanol | 0.00 | -0.64 |
|  | Naphthalene | 0.00 | -0.64 |
| Nocardioides | Styrene | 0.00 | 0.90 |
|  | β-Pinene | 0.00 | 0.77 |
|  | β-Myrcene | 0.00 | 0.94 |
|  | Caryophyllene | 0.00 | 0.99 |
|  | Decanal | 0.00 | 0.66 |
|  | Undecanal | 0.00 | 0.61 |
|  | Citral | 0.00 | 0.86 |
|  | Dodecanal | 0.00 | 0.85 |
|  | Linalool | 0.00 | 0.69 |
|  | Geraniol | 0.00 | 0.77 |
|  | 1-Nonanol | 0.00 | 0.85 |
|  | Naphthalene | 0.00 | 0.85 |
|  | D-Limonene | 0.00 | -0.91 |
|  | 3-Carene | 0.00 | -0.74 |
| Pseudokineococcus | Styrene | 0.00 | 0.88 |
|  | β-Pinene | 0.00 | 0.90 |
|  | β-Myrcene | 0.00 | 0.81 |
|  | Limonene | 0.00 | 0.83 |
|  | Cyclohexene | 0.00 | 0.81 |
|  | Caryophyllene | 0.00 | 0.88 |
|  | Citral | 0.00 | 0.85 |
|  | Dodecanal | 0.00 | 0.83 |
|  | Geraniol | 0.00 | 0.80 |
|  | 1-Nonanol | 0.00 | 0.83 |
|  | Naphthalene | 0.00 | 0.83 |
|  | D-Limonene | 0.00 | -0.73 |
|  | Nonanal | 0.00 | -0.65 |
